# Deep learning applied to EEG source-data reveals both ventral and dorsal visual stream involvement in holistic processing of social stimuli

**DOI:** 10.1038/s41598-023-34487-z

**Published:** 2023-05-05

**Authors:** Davide Borra, Francesco Bossi, Davide Rivolta, Elisa Magosso

**Affiliations:** 1 Department of Electrical, Electronic and Information Engineering “Guglielmo Marconi” (DEI), University of Bologna, Cesena Campus, Cesena, Italy; 2 MoMiLab Research Unit, IMT School for Advanced Studies Lucca, Lucca, Italy; 3 Department of Education, Psychology, and Communication, University of Bari Aldo Moro, Bari, Italy; 4 Alma Mater Research Institute for Human-Centered Artificial Intelligence, University of Bologna, Bologna, Italy

**Keywords:** Learning algorithms, Perception

## Abstract

Perception of social stimuli (faces and bodies) relies on “holistic” (i.e., global) mechanisms, as supported by picture-plane inversion: perceiving inverted faces/bodies is harder than perceiving their upright counterpart. Albeit neuroimaging evidence suggested involvement of face-specific brain areas in holistic processing, their spatiotemporal dynamics and selectivity for social stimuli is still debated. Here, we investigate the spatiotemporal dynamics of holistic processing for faces, bodies and houses (adopted as control non-social category), by applying deep learning to high-density electroencephalographic signals (EEG) at source-level. Convolutional neural networks were trained to classify cortical EEG responses to stimulus orientation (upright/inverted), separately for each stimulus type (faces, bodies, houses), resulting to perform well above chance for faces and bodies, and close to chance for houses. By explaining network decision, the 150–200 ms time interval and few visual ventral-stream regions were identified as mostly relevant for discriminating face and body orientation (lateral occipital cortex, and for face only, precuneus cortex, fusiform and lingual gyri), together with two additional dorsal-stream areas (superior and inferior parietal cortices). Overall, the proposed approach is sensitive in detecting cortical activity underlying perceptual phenomena, and by maximally exploiting discriminant information contained in data, may reveal spatiotemporal features previously undisclosed, stimulating novel investigations.

## Introduction

As human beings, we tend to use our faces to convey pieces of information during our interactions, e.g., emotional expressions or information related to identity, age or gender^1^. Specific neural structures^2,3^ are devoted to processing this information in a few hundred milliseconds^4–6^. We can process faces in a preferential way thanks to *holistic* perception: these perceptual mechanisms allow us to perceive the face as a whole (or *gestalt*), which is more than the sum of the single features composing it^7,8^. Holistic processing of faces was classically proven by the so-called *face-inversion effect* (FIE)^9^, i.e., upside-down faces are harder to perceive and identify than faces shown in the upright orientation. In particular, the picture-plane inversion of visual stimuli represents an ideal condition for vision research since the two conditions (upright vs. inverted) can be compared by controlling low-level visual properties of the image (e.g., luminosity, frequency, stimulus size)^10^.

Nevertheless, the human face is not the only type of stimulus we rely on during social interactions: the body also conveys pieces of information about the person we are interacting with. Indeed, several studies showed that bodies are affected by the inversion effect as well (i.e., *body-inversion effect*; BIE). Therefore, many researchers hypothesized that faces and bodies may share some common holistic processes^11–14^.

Both FIE and BIE have specific neurophysiological marks when assessed with various neuroimaging techniques, such as functional magnetic resonance imaging (fMRI) and electroencephalography (EEG). Traditional univariate fMRI analyses shows a network of bilateral cortical and subcortical brain regions that correlate with (upright) face perception; this network includes the fusiform face area (FFA)^15^, located in the fusiform gyrus, and the so-called occipital face area (OFA)^16^, located in the lateral occipital gyrus. In particular, FFA often shows reduced fMRI activity when faces are presented inverted (FIE), whereas OFA does not always show this effect, suggesting that FFA represents the strongest cortical representation of holistic processing^17^. Interestingly, inversion of face stimuli also engages other non-face areas such as the lateral occipital complex^18^, the precuneus^19^ and the intraparietal sulcus^20^, which are respectively involved in object processing^21^, mental rotation^22^ and visual working memory^23^. Analogously, fMRI also shows that body perception relies on a network of dedicated brain regions such as the extrastriate body area (EBA, located at the posterior end of the inferior temporal sulcus) and fusiform body area (FBA, in the fusiform gyrus)^24^. The EBA shows greater activation during perception of human bodies and body parts compared to objects or faces^25^, while the FBA responds to the form of the whole body rather than body parts^26^, thus showing a specific and separate function from EBA. Moreover, the FBA may distinguish between familiar and unfamiliar bodies^27^. Since BIE also involves face areas (i.e., OFA and FFA)^28^, it is likely that holistic processing for bodies might be mediated also by face-sensitive areas involved in head processing. In EEG, a negative Event-Related Potential (ERP) peaking around 170 ms after stimulus onset (N170) is consistently elicited by human faces^29–31^ and, sometimes with slightly later latency (around 190 ms post-stimulus), by bodies^32^. This component shows longer latency and often larger amplitude for inverted (compared to upright) faces^10,30,33^ and bodies^34^. The N170 is therefore thought to be generated by neural processes involved in structural encoding stages, where the representation of the configuration is created for recognition^29^.

Thus, the previous discussed fMRI and EEG studies indicate that holistic processing (for both faces and bodies) is a perceptual mechanism that occurs within 150–200 ms after the stimulus onset over face and non-face regions. One main limitation of the above reviewed “traditional” analyses of neuroimaging data is that they very often focus on a set of selected regions of interest (ROIs) in fMRI, and selected electrodes, time intervals or frequency bands in EEG studies; thus, the effects outside the “analysis lens” can be missed, increasing the risk of discarding potentially relevant (but unknown) neural features. A way to overcome these limitations, and thus gauge more information from neuroimaging data, is through an advanced application of machine learning. Furthermore, EEG studies are mainly focused on scalp-level analyses rather than source-level analyses (enabled via EEG source reconstruction); as an example, in our past study^35^, only scalp-level analyses were performed, neglecting the possibility of exploring time- and frequency-domain EEG changes associated to holistic processing in the different cortical regions.

Machine learning has gained increasing interest in neuroscience^36^ as a tool not only able to decode brain or behavioral states from multivariate time series, but also to shed light on the neural features underlying the decoded states^37^. In particular, deep learning approaches enable to explore almost entirely the information contained in neural signals in an automatic way, without relying on a priori, hand-crafted feature extraction. Specifically, convolutional neural networks (CNNs) have been recently adopted to decode and analyze neural time series both recorded invasively (i.e., electrocorticography—ECoG—and single-neuron recordings)^38,39^, and especially non-invasively (i.e., EEG)^40–48^, representing the principal deep learning approach applied to EEG^49^.

CNNs automatically learn the features that maximize between-class discriminability directly from the input multivariate neural activity and proved to significantly outperform traditional machine learning approaches (e.g., linear discriminant analysis or support vector machines applied on handcrafted EEG features) in a variety of tasks (e.g., motor and attention decoding)^46,47,50–52^. Thus, the features learned by CNNs, being automatically learned on the input and providing improved decoding capabilities, likely characterize the neural processes underlying the decoded states in a more complete and reliable way than traditional machine learning approaches and, even more complete than traditional EEG analyses. Therefore, visualizing and interpreting the features learned by the CNNs may offer great potentiality. In particular, a successful approach consists in combining CNNs with explanation techniques (ETs), such as saliency maps^53^ or layer-wise relevance propagation (LRP)^54^. ETs explain the network decision towards a specific decoded class (i.e., one brain or behavioral state) by identifying the input features inside an interpretable domain (spatial, temporal, or frequency domain) that mostly drive network decision^40–47^ and thus that are most discriminative of the specific decoded state. Hence, the combination CNN + ET provides a data-driven analysis tool (i.e., with no or minimum a priori assumption), able to provide insights into the neural correlates of the decoded brain or behavioral states.

The aim of the current study is to use a data-driven workflow based on the combination CNN + ET to analyze the inversion effect (i.e., holistic processing) of social stimuli i.e., face and body stimuli, using non-social (i.e., houses) stimuli as control. The analysis was conducted at source-level, by reconstructing the cortical activity from high-density EEG signals (HD-EEG) acquired during the presentation of social (faces and bodies) and non-social (houses) stimuli, displayed both upright and inverted. For each stimulus type (faces, bodies, houses), the reconstructed cortical signals were decoded via CNNs to discriminate between the two orientations. Then, a procedure based on an ET (here realized via LRP) was adopted to identify the brain regions most relevant for discriminating inverted vs upright orientation in social stimuli (faces and bodies) processing. Finally, analyses both in time domain [via event-related potentials (ERPs)], and in time–frequency domain [via event-related desynchronization/synchronization (ERD/ERS)] were applied to the so identified most relevant regions, to assess the different patterns of activity elicited in these areas by the two stimulus orientations.

We expected to find better performance of CNN classifiers for upright vs. inverted faces and bodies (given the specific markers for holistic processing in social stimuli) compared to houses. Moreover, we hypothesized that the proposed approach, by exploiting spatiotemporal patterns maximally discriminative for upright vs. inverted orientation, can not only support current knowledge about areas involved in holistic processing of social stimuli but also to disclose cortical regions so far overlooked, thus stimulating further experiments for deeper investigations.

## Materials and methods

Here, we first describe the EEG dataset used in this study. Then, we present the analysis workflow, based on the CNN + ET approach, developed to analyze the EEG data reconstructed at source level, identifying the cortical regions mostly involved in FIE and BIE. Lastly, time and time–frequency analyses performed on these cortical regions are described.

### Data description

**Figure 1:**
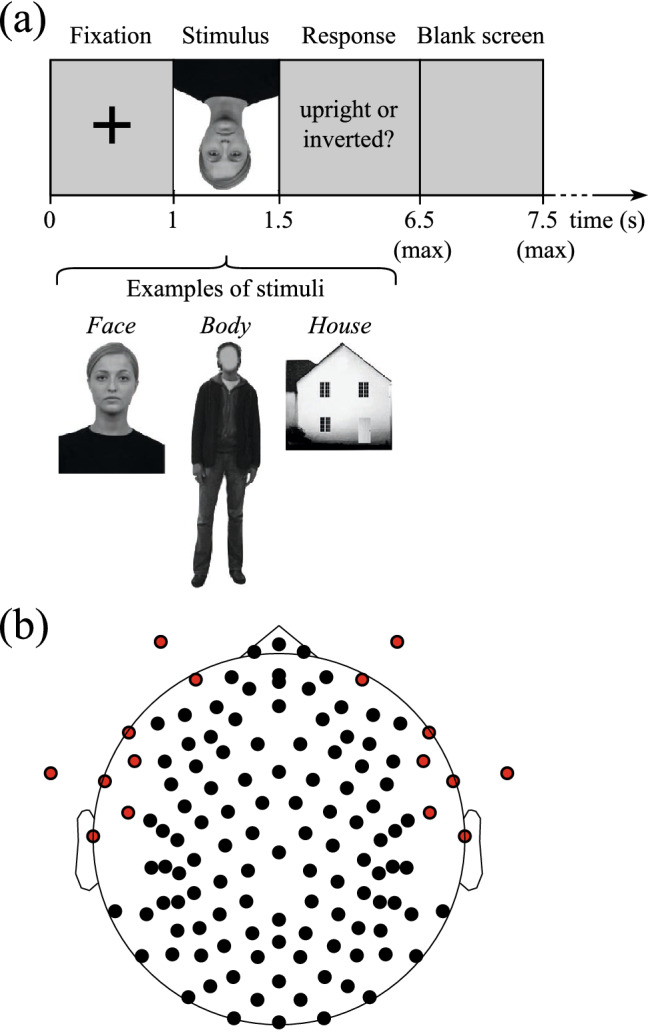
(**a**) Trial structure and examples of face, body and house stimuli. Each trial started with a fixation interval of 1 s. Then, a stimulus (a face, body, or house, presented with an upright or inverted orientation) was displayed for 500 ms. The participant had to identify whether the stimulus was presented with an upright or inverted orientation (response interval of up to 5 s). Lastly, the trial ended with an empty grey screen displayed for 1 s. The images of the face, body and house exemplary stimuli were taken from Bossi et al.^35^. (**b**) Locations of the 128 electrodes used in the high-density EEG recordings. Channels over ears and cheeks were removed from the analysis and are marked in red.

In this study, we used the signals recorded and analyzed at the scalp level by Bossi et al.^35^. Recordings were conducted at University of East London (UEL), and were approved by the local Ethic Committed of UEL, and conducted in accordance with the ethical standards laid out in the 1964 Declaration of Helsinki^35^. All participants gave written informed consent before enrolment in this study^35^. HD-EEG signals (128 channels) were recorded from 24 subjects [11 M; age of 28.2 ± 5.8 years, mean (m) ± standard deviation (std)] while looking at pictures of faces, bodies and houses in upright and inverted orientations. The experimental paradigm consisted in the presentation of 96 pictures to each subject, one picture per trial. Pictures contained face, body and house stimuli, in equal proportion, within each subject-specific dataset (i.e., 32 pictures per stimulus). Additional details about properties of stimuli and manipulations performed on stimuli can be found in our previous study^35^. Signals were recorded during three blocks of 32 trials each, with each block corresponding to a specific stimulus type. Block order was randomly permuted across subjects. For each stimulus type, half of pictures were presented to the subject upright, and the other half inverted, in a counterbalanced order within each block. Each trial began with an interval of 1 s, during which a fixation cross was shown. Then, the stimulus was presented for 500 ms (during which participants were explicitly instructed to not move their eyes or blink) and was followed by a response screen (lasting 5 s at most) during which the participant had to respond whether the stimulus was presented in an upright or inverted orientation, by pressing a button. After the response, a grey screen was presented for 1 s before triggering the next trial (Fig. 1a).

HD-EEG was recorded using a 128-channel Hydrocel Geodesic Sensor Net (Electrical Geodesic Inc., EGI, Eugene, OR, USA) referenced to the vertex^55^ (Fig. 1b); signals were amplified with an EGI NetAmps 400 amplifier and sampled at 1000 Hz, after application of a low-pass antialiasing filter. No other filter was applied during recording. Electrode impedances were kept below 50 kΩ.

### Data analysis

**Figure 2:**
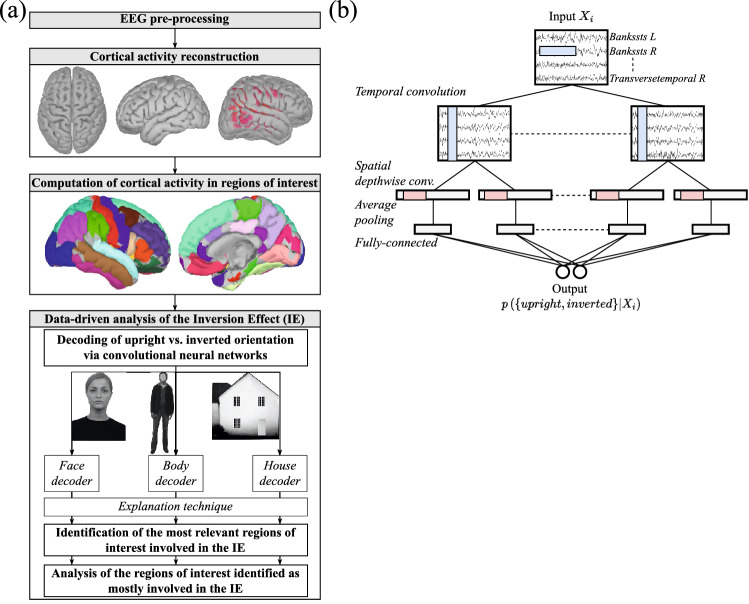
(**a**) Main stages of the data-driven analysis of inversion effect. High density EEG signals were first pre-processed, then the activity at cortical sources was estimated using sLORETA. Subsequently, the cortical surface was parcellated into regions of interest (ROIs) according to Desikan–Killiany atlas, and a single waveform representative of the activity of each ROI was derived. Then, a CNN-based decoder was applied to the activity of all ROIs for each single trial, to classify between upright and inverted orientation of the corresponding presented stimulus; note that this was done separately for trials relative to face, body, and houses, i.e., stimulus-specific neural decoders of upright vs. inverted orientation were designed. An explanation technique was applied to each decoder to quantify the relevance of each ROI for the classification, i.e., to quantify how much each ROI was important for discriminating the orientation. The images of the face, body and house exemplary stimuli were taken from Bossi et al.^35^. (**b**) High-level scheme of each CNN-based decoder. The neural decoder was based on a light and compact CNN. The CNN accepted as input a 2-D matrix (${X}_{i}$) containing the activities of all ROIs for a single trial (reporting ROIs by rows and time samples by columns) and provided as output the probability that the input cortical responses was related to an upright or inverted orientation of the eliciting stimulus ($p\left(\left\{upright,inverted\right\}|{X}_{i}\right)$). For clarity and brevity, only the main layers (i.e., convolutional and fully-connected layers) and pooling layers (changing temporal dimension) are represented in the scheme of the CNN. Black boxes represent the layer outputs, and internal colored rectangles represent convolutional (blue) and pooling (red) kernels. See Section S1 in the Supplementary Information for additional details about the neural network.

This section describes the data-driven analysis workflow. The main steps of the workflow are summarized in Fig. 2a.

#### EEG pre-processing

Data of one participant were discarded due to technical problems related to data quality; therefore, EEG signals from 23 participants were used. The same pre-processing performed in Bossi et al.^35^ was applied here, to extend our past analyses conducted on the scalp-level^35^ as fair as possible. Pre-processing was performed in MATLAB using the same version of the Toolbox EEGLAB^56^ (version 13_5_4b) as in our previous study^35^. It consists of the following stages:

1. Removal of channels on ears and cheeks (16 in total, see Fig. 1b), thus, resulting in 112 total channels.
2. Band-pass filtering between 1 and 100 Hz and notch filtering at 50 Hz.
3. Epoching into 2 s-length trials, starting from the presentation of the fixation cross and ending 500 ms after the presentation of the response screen. Thus, trials were defined from − 1 s to 1 s, where 0 s corresponds to the onset of the stimulus presentation (see Fig. 1a).
4. Baseline correction of each trial, by removing the mean value computed between − 1 s to 0 s, channel by channel.
5. Removal of bad epochs (87 ± 4 trials per subject after removal, m ± std) and of bad channels (23 ± 7 channels per subject removed, m ± std) by visual inspection.
6. Common average referencing.
7. Removal of ocular and muscular artifacts via independent component analysis (ICA).
8. Spherical spline interpolation of the bad channels removed in step 5.
9. Common average referencing (computed after bad channels interpolation, step 8).

#### Cortical activity reconstruction: source-level analysis

To transform sensor-space signals (scalp signals) into source-space signals (cortical signals), source localization was performed using the Brainstorm toolbox^57^ (version 3.220504) for MATLAB (version R2021a, The MathWorks, Inc.). A template head anatomy was adopted using the ICBM152 template, with the source space restricted to the cortex and discretized into 15002 vertices. The forward problem was solved using OpenMEEG^58^ and via the boundary element method (BEM), applying Brainstorm default values. The inverse problem was solved using sLORETA^59^, with identity noise covariance matrix, Brainstorm default regularization parameters (i.e., noise covariance regularization and signal-to-noise-ratio ($1/\lambda$) parameters set to 0.1 and 3, respectively), and free orientation of the sources, resulting in 3 directional components for each cortical vertex (i.e., 3·15002 source signals).

#### Computation of cortical activity within regions of interest (ROIs)

Following cortical activity reconstruction, the source-level activity contained in each trial was described by 3·15002 source signals, each lasting 2 s at 1000 Hz. Therefore, source-level activity was further processed with the aim of reducing data dimensionality. At first, signals were resampled at 200 Hz and trials were cropped in time between − 0.5 s and 0.5 s relative to stimulus presentation. Then, the cortical surface was parcellated into regions of interest (ROIs) using the Desikan–Killiany atlas^60^, which includes 68 ROIs ($R=68$) embracing the whole cortical surface (see Table 1 for the list of the ROIs), and, for each trial, a single waveform representative of the neural activity of each ROI was derived. To this aim, for each ROI, principal component analysis was applied to all signals of the vertices belonging to that ROI (i.e., to ${3\cdot N}_{r}$ features, denoting with ${N}_{r}$ the number of cortical vertices within the r-th ROI); then, the first principal component, explaining most of the variability of the data inside the ROI, was extracted as the signal representative of the entire ROI activity. The ambiguity on the sign of the first principal component was removed by imposing that its maximum had the same polarity as the maximum in the original feature that most contributed to the first component itself.

**Table 1:** List of the ROIs of the Desikan Killian Atlas [34 ROIs, both in left (L) and right (R) hemisphere]. The complete name of each ROI is reported on the right, while the corresponding abbreviation used in the figures is reported on the left.

ROI abbreviation	ROI name
*bankssts*	Banks superior temporal sulcus
*caudalanteriorcingulate*	Caudal anterior-cingulate cortex
*caudalmiddlefrontal*	Caudal middle frontal gyrus
*cuneus*	Cuneus cortex
*entorhinal*	Entorhinal cortex
*frontalpole*	Frontal pole
*fusiform*	Fusiform gyrus
*inferiorparietal*	Inferior parietal cortex
*inferiortemporal*	Inferior temporal gyrus
*insula*	Insular cortex
*isthmuscingulate*	Isthmus–cingulate cortex
*lateraloccipital*	Lateral occipital cortex
*lateralorbitofrontal*	Lateral orbital frontal cortex
*lingual*	Lingual gyrus
*medialorbitofrontal*	Medial orbital frontal cortex
*middletemporal*	Middle temporal gyrus
*paracentral*	Paracentral lobule
*parahippocampal*	Parahippocampal gyrus
*parsopercularis*	Pars opercularis
*parsorbitalis*	Pars orbitalis
*parstriangularis*	Pars triangularis
*pericalcarine*	Pericalcarine cortex
*postcentral*	Postcentral gyrus
*posteriorcingulate*	Posterior-cingulate cortex
*precentral*	Precentral gyrus
*precuneus*	Precuneus cortex
*rostralanteriorcingulate*	Rostral anterior cingulate cortex
*rostralmiddlefrontal*	Rostral middle frontal gyrus
*superiorfrontal*	Superior frontal gyrus
*superiorparietal*	Superior parietal cortex
*superiortemporal*	Superior temporal gyrus
*supramarginal*	Supramarginal gyrus
*temporalpole*	Temporal pole
*transversetemporal*	Transverse temporal cortex

#### Data-driven analysis of the stimulus inversion effect

##### Main concepts of the CNN + ET approach

We developed a CNN + ET approach to compute the relevance of each ROI and each time sample in discriminating between inverted vs. upright orientation, separately for each stimulus. The approach was applied to post-stimulus ROI activities, that is, to ROI signals from 0 to 0.5 s ($T=100$ time samples). Therefore, each trial was described by a 2-D matrix of shape $\left(R,T\right)=\left(\mathrm{68,100}\right)$, and was provided as input to the CNN + ET approach. Three datasets, one per stimulus type (faces, bodies, houses), were associated to each subject. For the generic subject $s$ (23 in total, with $0\le s\le 22$), the stimulus-specific dataset consisted of the collection of all ROI-level trials associated to that stimulus type and belonging to both orientation conditions i.e., upright and inverted; moreover, each trial was paired to a label indicating the orientation the trial was relative to. For simplicity, in describing the following concepts, we will refer to a single stimulus-specific dataset for each subject, since the CNN + ET procedure was replicated identically for each of the three datasets.

Let the subject-specific dataset (e.g., the face dataset) be denoted by:

1 $${D}^{(s)}=\left\{\left({X}_{0}, {y}_{0}\right),\dots ,\left({X}_{i}, {y}_{i}\right),\dots ,\left({X}_{{M}^{\left(s\right)}-1}, {y}_{{M}^{\left(s\right)}-1}\right)\right\},$$

where ${M}^{(s)}$ indicates the number of trials of this dataset for the s-th subject. ${X}_{i}$ represents the ROI activations in the i-th trial ($R$ signals at $T$ time samples) of the s-th subject and ${y}_{i}$ is the associated label among the ${N}_{c}=2$ possible classes, i.e., upright and inverted orientation (for brevity, in quantities ${X}_{i}$ and ${y}_{i}$ we omit the superscript of the subject index):

2 $$\left\{\begin{array}{c}{X}_{i}\in {\mathbb{R}}^{R\times T}, 0\le i\le {M}^{\left(s\right)}-1\\ {y}_{i}\in L=\left\{{l}_{0},{l}_{1}\right\}=\left\{\mathrm{upright},\mathrm{ inverted}\right\}.\end{array}\right.$$

The CNN can be trained to realize a classifier $f$ aimed to discriminate between the upright and inverted orientations of the stimulus presented to the subject. By doing so, the CNN automatically learns, from a training set of ROI-level trials, the most relevant features for a correct classification, so that it can subsequently detect inverted orientation on unseen trials belonging to the test set. Thus, the CNN describes the non-linear function $f$:

3 $$f\left({X}_{i};\theta \right):{\mathbb{R}}^{R\times T}\to L,$$

parametrized in the parameter array $\theta$ (whose values are learned during training) and mapping the single-trial neural activity contained in ${X}_{i}$ to a label. The knowledge learned by the function $f\left({X}_{i};\theta \right)$ to detect the inverted orientation is encoded in the parameter values $\theta$. Then, the CNN can be combined with an ET to identify the most relevant ROIs and temporal samples within the input ${X}_{i}$ that, based on the learned knowledge $\theta$, drive the decision of the CNN towards the inverted class. Specifically, for each ROI and temporal sample of the input trial ${X}_{i}$, the ET quantifies the importance of that ROI and time sample for the network to detect the inverted orientation. Thus, the ET provides a *relevance representation* $g$ of the input trial ${X}_{i}$:

4 $$g\left({X}_{i}\right): {\mathbb{R}}^{R\times T}\to {\mathbb{R}}^{R\times T}.$$

The function $g$ depends on the trained classifier $f$, on the network decision under investigation (e.g., inverted orientation) and on the specific explanation technique adopted to produce the relevance representation.

Accordingly, the CNN + ET approach consists of applying a non-linear transformation $g\left({X}_{i}\right)$ to the input trial ${X}_{i}$ to enhance, already at the single-trial level, meaningful characteristics contained in the neural activity (e.g., ROI activities), both in space and time, to distinguish the inverted vs. upright orientation. This approach, by exploiting automatically learned knowledge, may provide meaningful neural signatures about the processing of inverted vs. upright stimuli in a data-driven way. Crucially, by training classifiers $f\left({X}_{i};\theta \right)$ and applying $g\left({X}_{i}\right)$ separately for each stimulus-specific dataset, neural signatures related to inversion effect can be identified in a stimulus-specific manner, separately for faces, bodies and houses.

##### Decoding of upright vs. inverted orientation: CNN description and performance evaluation

For each stimulus-specific dataset, the CNNs were trained to discriminate between upright vs. inverted orientation of the presented stimulus, to learn the function in Eq. (3). The CNN structure was based on previous designs proposed in the literature for decoding event-related potential components (mainly P300 components) from single-trial EEG activity^45,50,52,61^. A peculiarity of the adopted design is its compactness, i.e., a limited number of parameters to fit (1502 in total, see Table S1 in Supplementary Information); this characteristic is important to avoid overfitting of small datasets, as the one adopted here. A schematic representation of the network is reported in Fig. 2b. First, the network processed the input neural activity ${X}_{i}$ using convolutions in time to learn how to optimally filter each ROI signal; then, convolutions in space were applied separately for each filtered version of the input (spatial depthwise convolution) to learn how to optimally combine the information across the different ROIs. Here, 4 different temporal kernels (i.e., 4 different filtered versions of the input), and 2 different spatial kernels for each filtered version of the input (thus, 8 in total) were learned. Then, a Rectified Linear Unit (ReLU) non-linear activation function was included to introduce the non-linearity in the model. Lastly, the output from the previous processing was downsampled in time (average pooling) to reduce the computational cost and was provided to a fully-connected layer with ${N}_{c}=2$ output neurons, one per class (i.e., upright and inverted). The activations of the two output neurons represented the output class-scores ${o}_{k}, k=\mathrm{0,1}$; these were passed through a softmax activation function to convert each output class-score in the probability that the input trial contains a neural response to an upright or an inverted stimulus, i.e., $p\left(\left.{l}_{k}\right|{X}_{i}\right), k=\mathrm{0,1}$. To increase the generalization of the model, batch normalization^62^ was added after each convolutional layer, and dropout^63^ was included immediately before the fully-connected layer. A more detailed description of the parameters defining the CNN structure is provided in Section S1 and in Table S1 in Supplementary Information.

The CNNs were trained to distinguish upright vs. inverted orientation separately for face, body, and house stimuli, resulting in three independent groups of stimulus-specific decoders. Therefore, the parameters contained in $\theta$ were optimized separately for the different stimuli. Specifically, for each type of stimulus (faces, bodies, and houses), a leave-one-subject-out cross-validation scheme was adopted: the stimulus-specific dataset of the s-th subject was held out and used as test set, while the stimulus-specific datasets of all other 22 subjects were used as training set. In addition, 10% of trials belonging to the training set were held out to form a validation set devoted to defining a stop criterion for the optimization (see later). All sets were standardized before network training, by using the mean value and standard deviation computed on training examples^46,64,65^. Furthermore, for each held-out subject (i.e., each cross-validation fold), the parameters ($\theta$) of the decoder were trained using different initializations. Indeed, different initialization points can cause the training procedure to end into different optimal sets of the parameters. To increase the robustness of the decoder to different initial values, it was trained 10 times by adopting 10 different seeds for the random initialization of the parameters, randomly sampled. For each type of stimulus, this led to 10 decoders trained for each held-out subject and since the procedure was repeated for each subject selected as held-out subject, a total of 230 (= 23 · 10) CNN decoders were trained for faces, bodies, and houses, separately (overall, 690 decoders). Details about the network training (e.g., optimization of the parameters $\theta$) are reported in Section S1 in the Supplementary Information. Table 2 contains a summary of the dataset properties, including the number of training, validation, and test examples.

**Table 2:** Dataset properties. The number of training, validation and test examples are reported as mean value ± standard deviation across leave-one-subject-out cross-validation folds.

Dataset property		Value
No. of decoded classes (${\mathrm{N}}_{\mathrm{c}}$)		2
No. of time steps (T)		100
No. of ROIs (R)		68
No. of examples	Training	573 ± 9
	Validation	67 ± 2
	Test	29 ± 2

As concerning the performance metrics, for each stimulus-specific decoder and each cross-validation fold we computed the confusion matrix and decoding accuracy, using the independent test set of the specific fold (i.e., the data of the held-out subject of that fold). Then, these metrics were averaged across the different random initializations (10 different seeds), resulting in an average metric for each fold of the leave-one-subject-out cross-validation.

Differences in decoding accuracies among the different stimulus-specific decoders were tested by performing pairwise comparisons, considering all possible combinations (3 tests). Wilcoxon signed-rank tests were used and were corrected for multiple tests using the Bonferroni correction. These comparisons were useful to potentially highlight different capabilities of the neuroelectric activity in discriminating upright vs. inverted orientation depending on the type of stimuli, evidencing different brain processing and encoding of the stimuli.

##### Explanation technique (ET) and identification of the ROIs mostly relevant for discrimination

In this study, layer-wise relevance propagation^54^ (LRP) was adopted as ET to realize the transformation in Eq. (4). LRP is a backward propagation technique, specifically designed for explaining deep neural networks, and operates by propagating the class score provided by the network (i.e., the output of the fully-connected layer immediately before the softmax activation) back to a target layer under investigation, using local propagation rules applied at each layer forming the neural network. The obtained relevance representation contains both positive and negative values, associated to positive and negative evidence for the model prediction. LRP was successfully applied to time series, e.g., to identify EEG patterns that explain decisions^40–42^. Theoretical details about LRP can be found in Section S2 of Supplementary Information.

In this study, LRP was used to obtain a relevance representation about the discrimination between inverted vs. upright stimuli in the temporal and spatial domains of the input (i.e., the propagation was terminated at the input layer), quantifying how much each input spatiotemporal feature contributed to the inverted class prediction. Specifically, the following processing was applied separately for each stimulus category (faces, bodies and houses). For each trained stimulus-specific decoder, we considered the trials corresponding to the stimuli with inverted orientation inside the independent test set of each cross-validation fold (i.e., for each held-out subject of the leave-one-subject-out partitioning scheme). For each of these trials provided as input to the neural decoder, the class-score associated to the inverted condition ${o}_{1}$ was backward propagated up to the input layer, using the $\varepsilon$-rule^66^ (see Section S2 in Supplementary Information for further details). Therefore, for each stimulus-inverted trial, a 2-D map having the same shape as the input (i.e., $\left(R,T\right)=\left(\mathrm{68,100}\right)$) was obtained, quantifying the relevance of each input feature in time and space for predicting the inverted class. These 2-D maps were first averaged across trials, and then across the different random initializations (10 different seeds), obtaining one *inversion relevance map* for each fold of the leave-one-subject-out cross-validation (i.e., 23 2-D representations in total).

Lastly, from the previous map, for each ROI ($R=68$ ROIs, overall), the maximum value within the 150–200 ms interval was extracted. The choice of this interval is justified since from the relevance maps it automatically resulted as the one most relevant for the discrimination, and it is the time interval in which the N170 component typically occurs^67,68^, with this component being modulated (larger and delayed peak) by inverted faces and bodies relative to upright^30,34,69^. The choice of considering the maximum value for each ROI within this interval is justified as we searched for ROIs that provided most evidence for the inverted class score (associated to more positive values for LRP computed from ${o}_{1}$). Following this procedure, within each cross-validation fold, each ROI was characterized by a single *inversion relevance score* (*RS*) (i.e., for each ROI, 23 scalar values were obtained, across folds).

**Figure 3:**
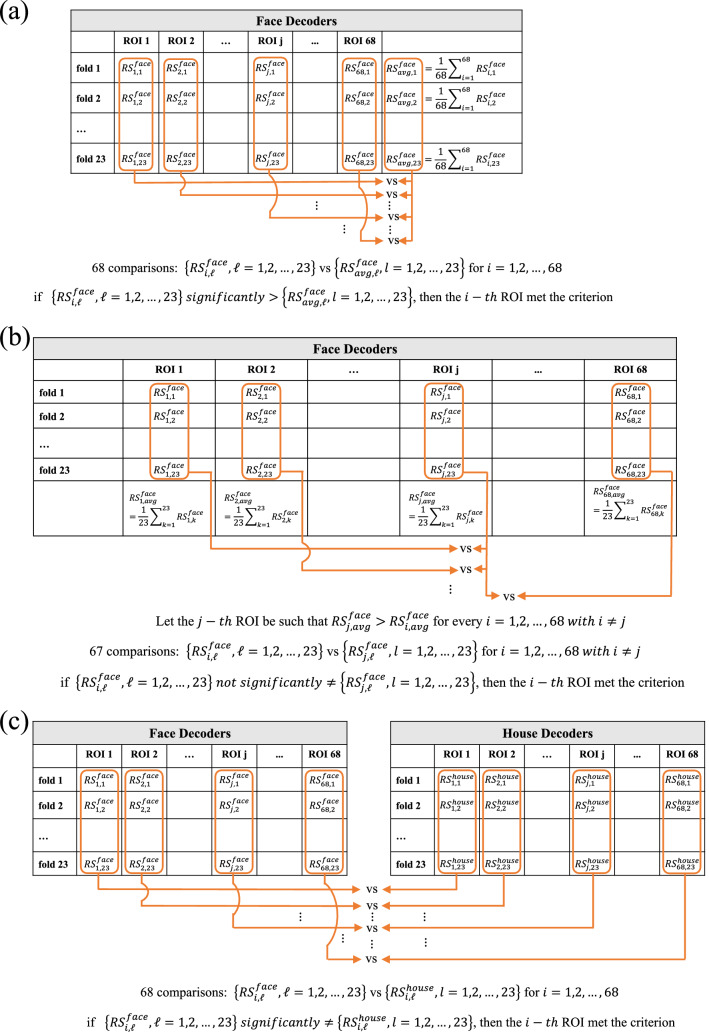
Criteria used for identifying the cortical ROIs most relevant for the network decision, i.e., that most contributed to the classification of inverted vs. upright orientation in case of social stimuli (i.e., faces and bodies). The figure is relative to face stimuli, but the same applies to body stimuli, by replacing face decoders with body decoders. ${RS}_{i,\mathcal{l}}$ is the inversion relevance score for the i-th ROI in the $\mathcal{l}$-th cross-validation fold, obtained as the maximum relevance value of the i-th ROI in the interval 150–200 ms, inside the inversion relevance map associated to the $\mathcal{l}$-th fold. (**a**) Criterion 1. Each ROI resulting to have relevance scores (23 values, one per fold) significantly higher than the average relevance scores across ROIs (23 values, one per fold) met the criterion. (**b**) Criterion 2. The ROI having maximum average relevance score across the folds was identified (e.g., the j-th ROI). Each ROI resulting to have relevance scores (23 values, one per fold) not significantly different from the relevance scores of that ROI (23 values, one per fold) met the criterion, together with that ROI. (**c**) Criterion 3. Each ROI having relevance scores (23 values, one per fold) significantly different from the relevance scores in case of house stimuli (23 values, one per fold), met the criterion. Only ROIs that met all three criteria were identified as most relevant.

Then, based on the so obtained relevance scores, we identified the ROIs mostly discriminant of inverted vs. upright orientation in social stimuli (i.e., faces and bodies), as the ones that met the following three “selection criteria” (see Fig. 3, schematizing criterion 1, 2, 3 respectively in Fig. 3a–c). These three criteria were assessed for the ROIs’ relevance scores derived from face decoders, and for those derived from body decoders, in a separate manner.

- *Criterion 1.* For each fold, we computed the average relevance score across all ROIs, obtaining an average relevance score. Then, we performed statistical pairwise comparisons between the resulting average relevance scores (23 samples) and the relevance scores (23 samples) of each ROI, for a total of 68 comparisons. Criterion 1 was met by the ROIs having relevance scores significantly higher than the average relevance scores, according to the statistical comparisons (Fig. 3, left upper panel).
- *Criterion 2.* At first, we identified the ROI with maximum relevance score (on average across the 23 folds) among the 68 considered ROIs. Then, we performed statistical pairwise comparisons between the relevance scores (23 samples) of that ROI (the ROI with maximum relevance) and the relevance scores (23 samples) of each of the other ROIs (68–1 = 67 remaining ROIs), for a total of 67 comparisons. Criterion 2 was met by the ROI having the maximum relevance and by all other ROIs comparable to it (i.e., not significantly different from it according to the statistical comparisons, Fig. 3, right upper panel).
- *Criterion 3*. For each ROI, we performed statistical pairwise comparisons between the relevance scores (23 samples) of that ROI in case of a social stimulus (faces and bodies, separately) and the relevance scores (23 samples) of the same ROI in case of the non-social (control) stimulus (houses), for a total of 68 comparisons. Criterion 3 was met by the ROIs having relevance scores in case of a social stimulus significantly different compared to the control stimulus (see Fig. 3, lower panel).

All previous pairwise comparisons were performed using Wilcoxon signed-rank tests. Bonferroni correction was used to correct for multiple tests, and significance alpha level was set to 0.05.

Thus, the ROIs identified as the mostly relevant for discriminating inversion of a social stimulus (face or body) were those having relevance scores for the inverted prediction: (1) superior to the average score across the ROIs, (2) comparable to the highest score, and (3) significantly different from the score obtained in case of the non-social stimulus.

##### Analysis of the most relevant ROIs

Once the ROIs mostly relevant in driving CNNs decision were identified according to the procedure above, we examined differences in activity pattern of these ROIs between upright and inverted stimulus orientation, separately for face and body stimuli. Indeed, as these ROIs mainly drove network decision, they likely exhibited differential patterns in the upright vs. inverted conditions. The examined ROI activity was the one obtained via source reconstruction (see “Computation of cortical activity within regions of interest”), i.e., the same activity given as input to the CNN + ET approach. The following analysis in the time domain and in the time–frequency domains was performed, for both face and body stimuli.

As to the *time-domain* analysis, for each subject, we computed the ERP of each identified ROI by averaging the ROI activity across trials, separately for the upright and inverted conditions. As to the *time–frequency analysis*, for each trial, the continuous wavelet transform of the ROI activity was computed using complex Morlet wavelet as basis function and transform coefficients were squared to obtain time–frequency power representations. Note that the obtained time–frequency representation referred to the ROI mixed activity, i.e., including both evoked and induced activity, so to characterize the overall activity processed by the CNN + ET approach, in the time–frequency domain. Then, these representations were normalized using the interval between − 0.5 s and 0 s as baseline, to compute ERD/S. Specifically, for each frequency, the average power value was computed across time samples between − 0.5 and 0 s obtaining the baseline value at that frequency; then, ERD/S were obtained as the difference between the time–frequency power values and the baseline value at the same frequency, divided by the baseline. Finally, for each subject, the ERD/S representations of each identified ROI were averaged across trials, separately for the upright and inverted conditions.

To evince differences between upright and inverted conditions in ERPs and ERD/S representations, permutation cluster tests (5000 iterations) with significance level at 0.05 were performed between the two conditions in the time and time–frequency representations.

## Results

### Decoding performance of CNNs

**Figure 4:**
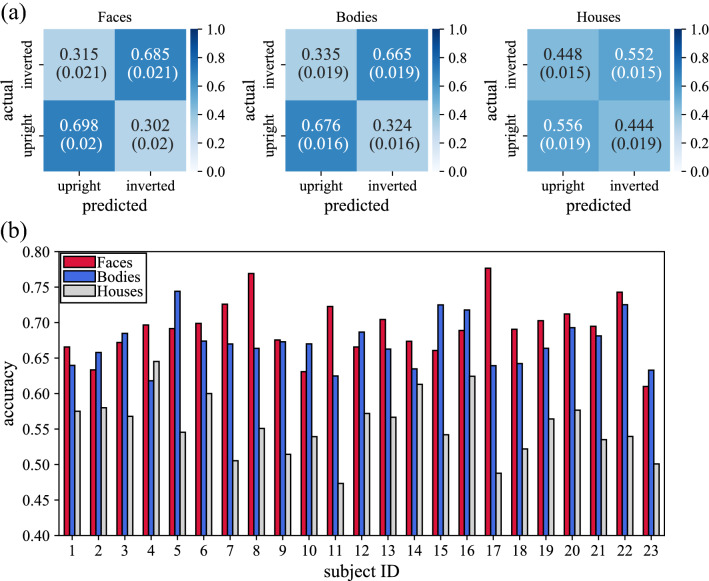
Performance metrics of CNNs discriminating upright vs. inverted orientation. (**a**) Confusion matrices. Mean values ± standard error of the mean (within brackets) of confusion matrices across subjects (i.e., across cross-validation folds) are reported, separately for each stimulus-specific (face, body, and house) decoders. (**b**) Accuracies. The accuracy scored on each subject (i.e., each cross-validation fold) is reported, separately for each stimulus-specific (face, body, and house) decoders.

For each stimulus-specific decoder discriminating between upright and inverted stimulus orientations, Fig. 4a reports the confusion matrices and Fig. 4b the decoding accuracies; the latter are reported subject-by-subject (i.e., for each fold of the leave-one-subject-out cross-validation).

Decoding accuracies were 0.692 ± 0.008, 0.671 ± 0.007, 0.554 ± 0.009 (mean ± standard error of the mean across folds), respectively for face, body, and house decoders. House decoders performed nearly at, but significantly above ($p<0.001$), the chance level. Face and body decoders obtained significantly higher accuracies than house decoders ($p<0.001$, in both cases), while no significant difference emerged between face and body decoders ($p>0.05$). It is worth reminding that for each fold, each performance metric was averaged across 10 randomizations, to provide a more robust evaluation of the decoder (thus, to reduce the dependency of the result on the specific random seed). The standard error of the mean of the decoding accuracy across randomizations varied between 0.006 and 0.015 across the 23 cross-validation folds and stimuli (face, body, house).

### Identification of the ROIs mostly relevant for discriminating orientation

**Figure 5:**
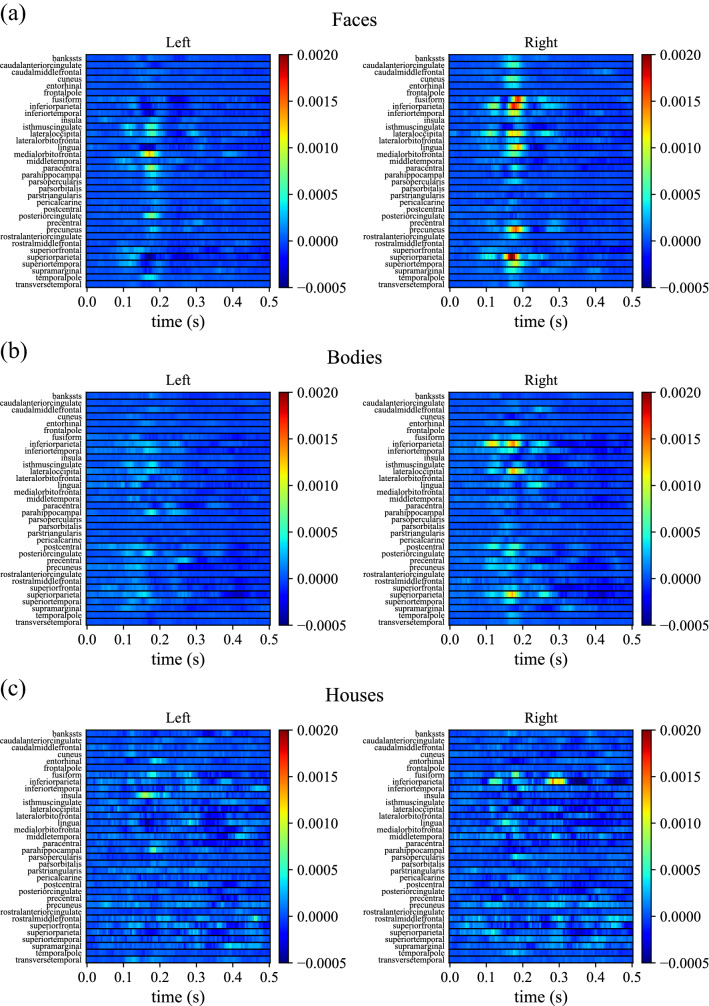
Inversion relevance maps across ROIs and time (after stimulus presentation) for predicting the inverted orientation in case of faces, bodies, and houses, reported in panels (**a**) to (**c**), respectively. The average relevance map across subjects (i.e., across cross-validation folds) is reported, separately for each hemisphere. Each of these 2-D heatmaps contains in each row the relevance over time for each specific ROI in discriminating inverted vs. upright orientation in case of face, body and house stimuli, respectively. For the sake of clarity, the relevance maps relative to cortical ROIs in the left and in the right hemisphere are displayed separately. In each map, horizontal black lines delimitate different ROIs. To shorten ROI names abbreviations were used; see Table 1 for the association between ROI abbreviations and names.

Figure 5 shows the relevance maps of neural activity for predicting inverted orientation in case of face, body, and house stimuli. The maps are averaged across subjects (i.e., across cross-validation folds) and displayed as heatmaps, separately for the left and right hemispheres.

The relevance values resulted higher in the right hemisphere. Furthermore, the time samples within the 150–200 ms interval (where the N170 component occurs) showed the highest importance in discriminating inverted orientations in case of social stimuli (faces and bodies), although the importance was highly modulated across ROIs, with few ROIs exhibiting much larger relevance than all the others in this time interval. Interestingly, the relevance values in this interval were lower in case of bodies than faces. On the contrary, the relevance maps in case of house stimuli appeared less defined in time and space (it is notable only a higher relevance in right inferior parietal cortex at about 300 ms post-stimulus), suggesting that only slight differences in neural activations were elicited by inverted vs. upright stimulus orientation, in line with accuracy close to chance level.

**Figure 6:**
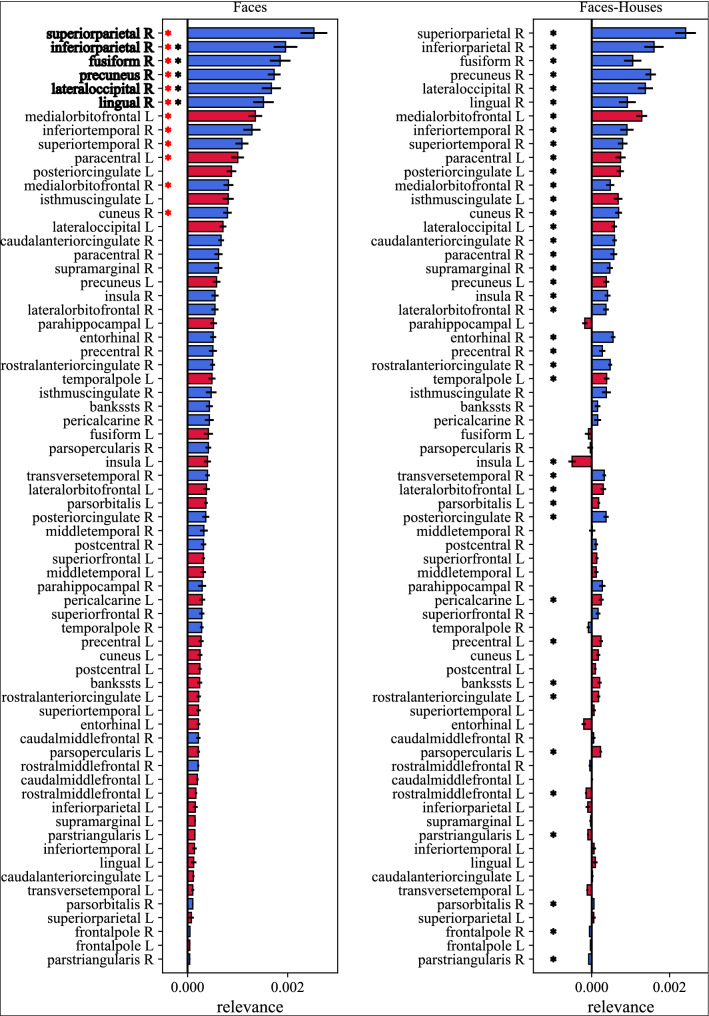
Inversion relevance scores of ROIs for predicting inverted faces. To compute the relevance scores, for each 2-D relevance representation of each subject (i.e., of each cross-validation fold), the maximum value within 150–200 ms was extracted, separately for each ROI. The left panel reports the so computed relevance scores for faces, while the right panel reports the difference between faces and houses. Bar heights and error bars denote the mean value and standard error of the mean across subjects (i.e., across cross-validation folds), respectively. Blue bars are associated to ROIs of the right hemisphere (R), while red bars to the ones of the left hemisphere (L). To shorten ROI names abbreviations were used; see Table 1 for the association between ROI abbreviations and names. Results of the performed statistical analyses are reported too. In the left panel, red asterisks denote ROIs having significantly higher relevance scores (*p* < 0.05) than the average relevance across ROIs (criterion 1); black asterisks denote ROIs having comparable relevance scores (*p* > 0.05) as the most relevant ROI (superior parietal R, criterion 2). In the right panel, black asterisks denote ROIs having significantly different relevance scores (*p* < 0.05) between faces and houses (criterion 3). All tests were Wilcoxon signed-rank tests corrected for multiple tests with Bonferroni correction. In the left panel, the most relevant ROIs resulting from all selection criteria are displayed in bold.

**Figure 7:**
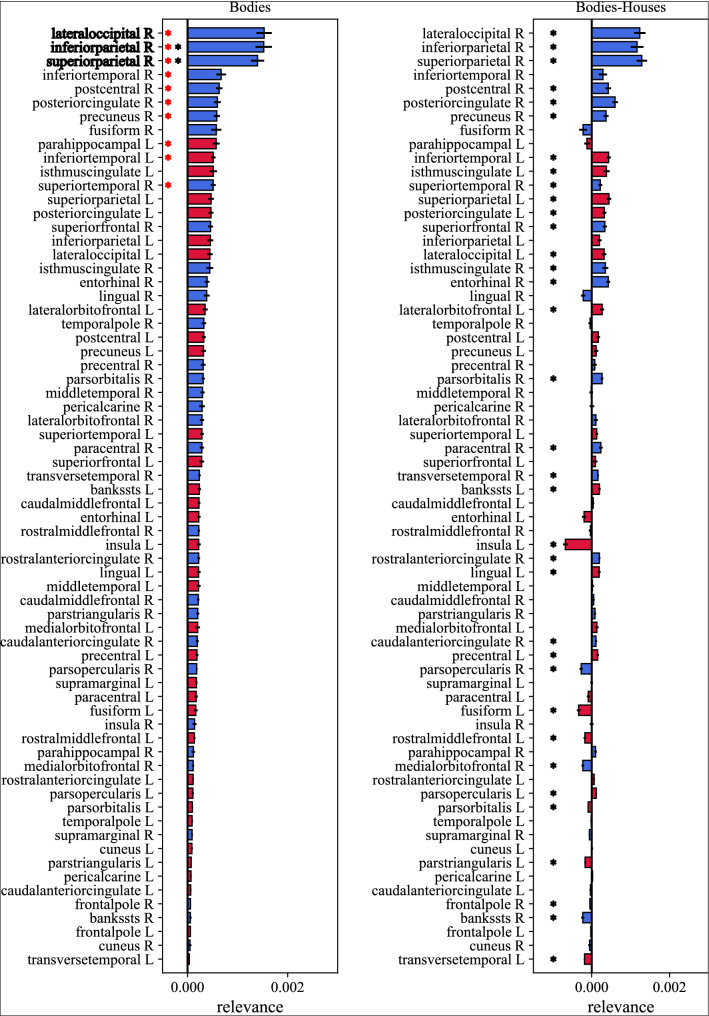
Inversion relevance scores of ROIs for predicting inverted bodies. To compute the relevance scores, for each 2-D relevance representation of each subject (i.e., of each cross-validation fold), the maximum value within 150–200 ms was extracted, separately for each ROI. The left panel reports the so computed relevance scores for bodies, while the right panel reports the difference between bodies and houses. Bar heights and error bars denote the mean value and standard error of the mean across subjects (i.e., across cross-validation folds), respectively. Blue bars are associated to ROIs of the right hemisphere (R), while red bars to the ones of the left hemisphere (L). To shorten ROI names abbreviations were used; see Table 1 for the association between ROI abbreviations and names. Results of the performed statistical analyses are reported too. In the left panel, red asterisks denote ROIs having significantly higher relevance scores (*p* < 0.05) than the average relevance across ROIs (criterion 1); black asterisks denote ROIs having comparable relevance scores (*p* > 0.05) as the most relevant ROI (lateral occipital R, criterion 2). In the right panel, black asterisks denote ROIs having significantly different relevance scores (*p* < 0.05) between bodies and houses (criterion 3). All tests were Wilcoxon signed-rank tests corrected for multiple tests with Bonferroni correction. In the left panel, the most relevant ROIs resulting from all selection criteria are displayed in bold.

The relevance maps were exploited to compute the relevance scores, used to rank up ROIs by their importance in discriminating inverted vs. upright orientations in social stimuli. These scores are reported in Figs. 6 and 7, for faces and bodies respectively (left panels), with ROIs sorted by their relevance in predicting the inverted orientation. Furthermore, for each ROI, the right panels in Figs. 6 and 7 also report the difference of the relevance scores between social (faces and bodies) and non-social (houses) stimuli, to better highlight how much each ROI differently processed the inverted orientation in case of social vs. non-social stimuli in the 150–200 ms post-stimulus interval. These figures also display the results of the statistical comparisons performed to identify the most discriminant ROIs in case of face stimuli and of body stimuli, highlighting a significant different of the relevance score, widely across ROIs, between social stimuli and non-social (control) stimuli, as qualitatively emerged from Fig. 5.

The most relevant ROIs, as identified by the selection criteria presented in Section “Explanation Technique (ET) and identification of the ROIs mostly relevant for discrimination” (with Criterion 3 resulting little selective), all belonged to the right hemisphere. These were, for *faces*: the superior parietal cortex, inferior parietal cortex, fusiform gyrus, precuneus, lateral occipital cortex and lingual gyrus; for *bodies*: lateral occipital cortex, inferior parietal cortex and superior parietal cortex. Interestingly, the three ROIs resulting most relevant for discriminating body orientation were also among the six most relevant ROIs for face orientation.

### ERPs and ERD/S in the ROIs identified as mostly relevant for discriminating orientation

**Figure 8:**
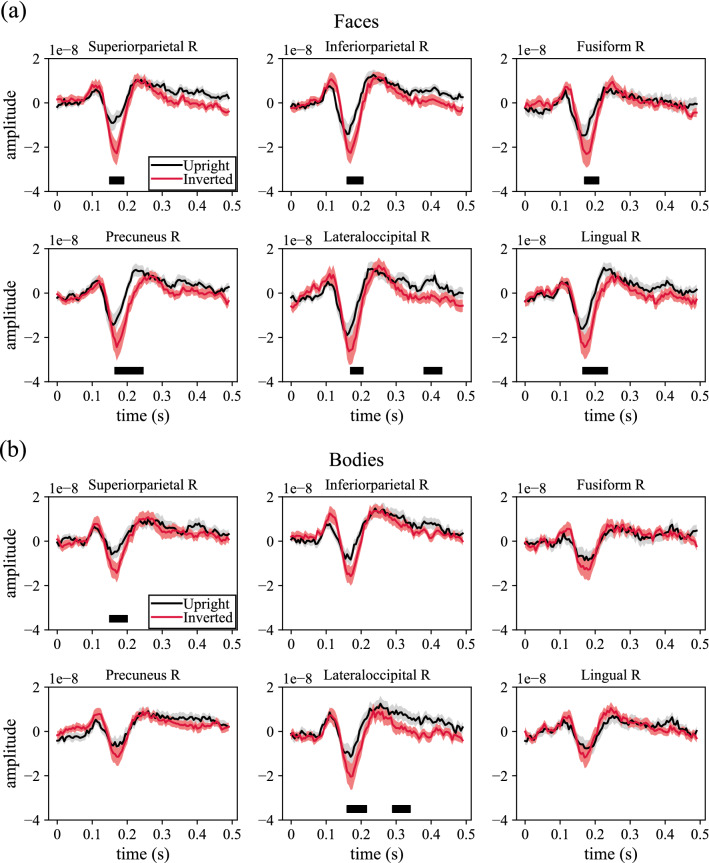
ERPs corresponding to face stimuli (**a**) and to body stimuli (**b**) computed for the 6 ROIs identified as the most discriminant of inverted vs. upright orientation across social stimuli (both faces and bodies). In each plot the ERP for upright (black) and inverted (red) conditions are reported. The mean value is reported as a thick line, while the standard error of the mean (across subjects) is reported as an overlayed area. To shorten ROI names abbreviations were used; see Table 1 for the association between ROI abbreviations and names. Results of the performed statistical analyses are reported too. Thick horizontal black lines on the bottom of each plot denote time intervals in which the ERPs associated to upright and inverted stimuli were significantly different (*p* < 0.05), as resulting from the performed permutation cluster tests.

Figure 8 reports the ERPs associated to the upright and inverted conditions of face stimuli (panel a) and body stimuli (panel b), and computed for the ROIs identified as mostly relevant for orientation discrimination. The ERPs of all the most relevant ROIs across social stimuli are reported (6 ROIs, for both face and body stimuli).

Inverted orientation produced a more pronounced N170 compared to upright orientation for both categories of social stimuli. The responses to upright and inverted orientations started to be significantly different from circa 150–170 ms and continued up to circa 250 ms after stimulus presentation, as a consequence of an increased latency in the response to the inverted orientation compared to the upright orientation (e.g., see the precuneus area for faces in Fig. 8a).

**Figure 9:**
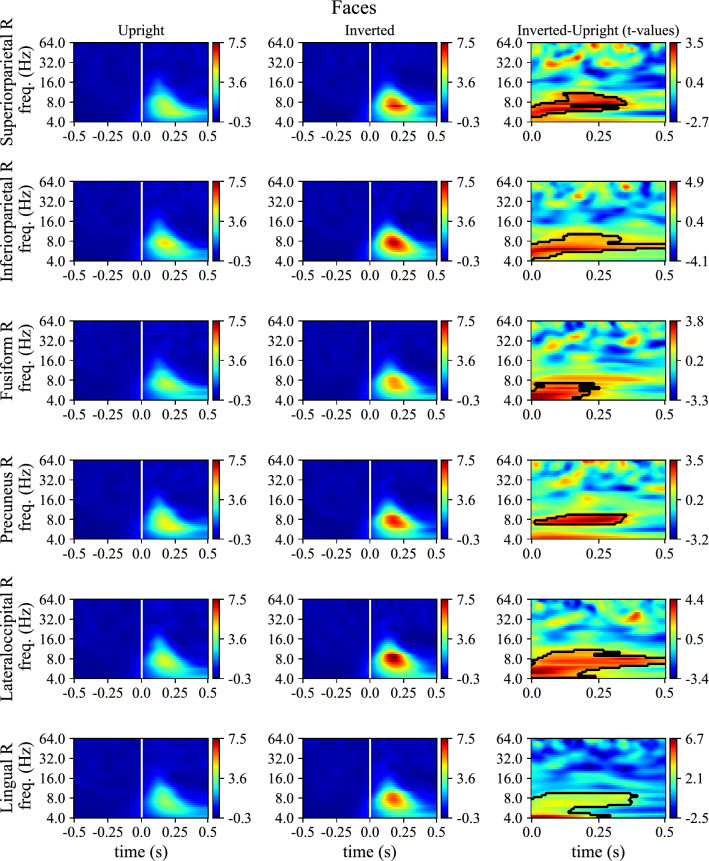
ERD/S of the mixed activity in case of upright and inverted face stimuli for the 6 ROIs identified as the most discriminant of inverted vs. upright orientation across social stimuli (both faces and bodies). For each ROI, ERD/S are displayed as heatmaps for upright and inverted conditions. Thick vertical white lines denote the onset of the presentation of stimuli (0 s). To shorten ROI names abbreviations were used; see Table 1 for the association between ROI abbreviations and names. Lastly, t-values obtained by comparing inverted to upright conditions via permutation cluster test are reported too (only post-stimulus); significant clusters (p < 0.05) are marked with thick black curves.

**Figure 10:**
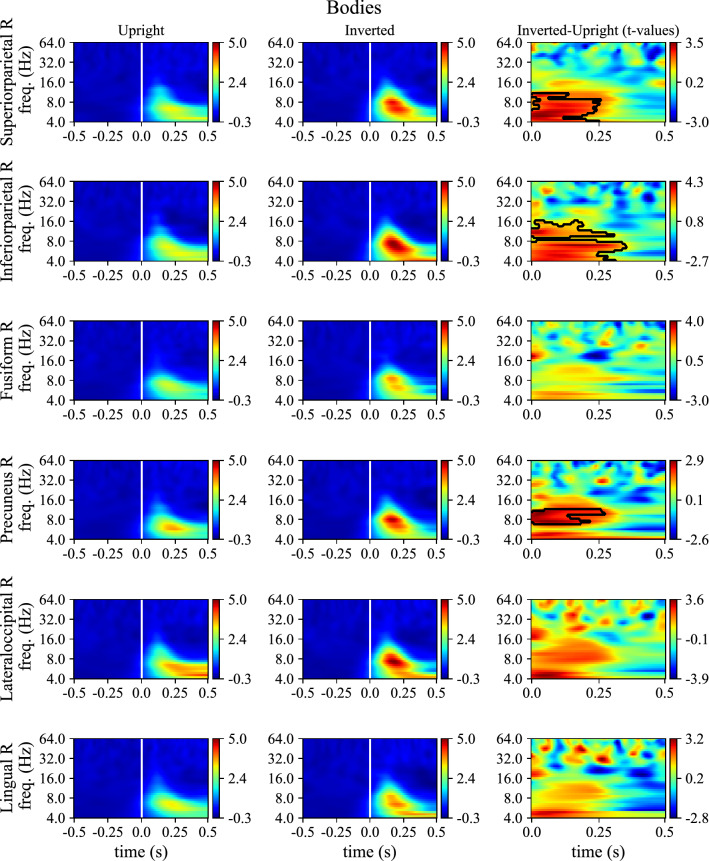
ERD/S of the mixed activity in case of upright and inverted body stimuli for the 6 ROIs identified as the most discriminant of inverted vs. upright orientation across social stimuli (both faces and bodies). For each ROI, ERD/S are displayed as heatmaps for upright and inverted conditions. Thick vertical white lines denote the onset of the presentation of stimuli (0 s). To shorten ROI names abbreviations were used; see Table 1 for the association between ROI abbreviations and names. Lastly, t-values obtained by comparing inverted to upright conditions via permutation cluster test are reported too (only post-stimulus); significant clusters (p < 0.05) are marked with thick black curves.

Lastly, Figs. 9 and 10 reports the mixed ERD/S, averaged across subjects, for upright and inverted conditions together with the *t*-values associated to the difference between the two conditions, for faces and bodies, respectively. Note that, like ERP representations (Fig. 8), ERD/S of all the most relevant ROIs across social stimuli are reported (6 ROIs, for both face and body stimuli).

A significantly increased synchronization in theta and alpha bands was found in inverted conditions. This occurred not only in early post-stimulus intervals in which the N170 potential occurs (i.e., before 200 ms), but also in later post-stimulus interval (in particular, see lateral occipital cortex, lingual gyrus, and inferior parietal cortex in Fig. 9 among the most relevant ROIs for face stimulus inversion).

## Discussion

Human face perception relies on holistic perceptual mechanisms, which can be disrupted by stimuli inversion (FIE)^9^; a similar phenomenon was observed with different social stimuli, i.e., bodies (BIE)^14^. In this study, we investigated the FIE and BIE as reconstructed from the scalp HD-EEG, by adopting a totally novel data-driven approach that implements a CNN combined with ET. To the best of our knowledge, this is the first time that a deep learning approach is used to analyze EEG activity related to FIE and BIE and that a CNN + ET method is applied at source-level rather than at scalp-level. As such, our study provides a double contribution: (i) aid the investigation of the neural basis of social stimuli processing, highlighting different and shared mechanisms between FIE and BIE in the temporal, spatial and frequency domains (contribution to the neurophysiological knowledge); (ii) propose an innovative approach based on explainable artificial intelligence (CNN + ET) that may have a more general validity in exploring event-related electrophysiological responses at source-level, being able to automatically discover the most relevant cortical features underlying the examined task with no or minimal a priori selection (methodological contribution). Specifically, these contributions allow to overcome the main limitations of our past study^35^ in which only scalp-level analyses were performed and based on some a priori selections (time intervals and frequency bands), by proposing and applying here (on the same data recorded in Bossi et al.^35^) a novel machine learning-empowered computational tool that automatically reveals the most relevant cortical regions for stimuli inversion to guide analyses at the source level, performed without a priori selection in the space, time and frequency domain.

Since the analysis was based on the prediction of the CNN-based decoders, the latter were first validated by examining their performance. The first relevant result is that high accuracies (well above the chance level) were obtained for face and body stimuli decoders discriminating between the two orientations, at variance with the control stimuli (houses) decoder, which on the contrary, performed close to chance. This confirms that faces and bodies are actually special stimuli processed holistically^70^, and that stimuli inversion alters these processes also at a neural level^33,34^. This different kind of (intact or disrupted) processing is learned and encoded by the CNNs for faces and bodies, but not or minimally for houses, which do not rely on holistic processing. Based on these results, CNNs could have captured useful neural features that properly characterize holistic processing when social stimuli are presented upright, vs. the disruption of these features or the recruitment of other neural features related to non-holistic processes (e.g., object-general processing systems) when social stimuli are presented inverted. Thus, we took advantage of the knowledge learned and encapsulated in the face and body decoders to study the spatiotemporal features of the cortical activity mostly relevant for orientation discrimination, and therefore that can be differentially implicated in holistic vs. non-holistic processing.

The analysis performed via the ET allowed the quantification of the relevance of different cortical ROIs and time samples for decoding face inversion and body inversion (Fig. 5). The 150–200 ms interval, where the N170 components appear, automatically emerged as crucial for detecting the inverted orientation. Moreover, the most relevant ROIs for both faces and bodies were the right superior parietal cortex, inferior parietal cortex, lateral occipital cortex, and for faces only, the right lingual gyrus, fusiform gyrus, and precuneus. Overall, the right hemisphere resulted more relevant than the left hemisphere in decoding face and body inversion. Taken together, these results identify a main time interval and a few cortical ROIs (mainly in the right hemisphere), where neural activity better discriminated between inverted vs. non inverted stimuli. It is important to note that some of the areas identified as most relevant are already known as being specifically involved in face perception and body perception (e.g., the lateral occipital cortex and fusiform gyrus) and to exhibit different activations in case of inverted compared to upright stimuli^13,26^. This represents a sort of validation of the proposed deep learning-based method, used here for the first time in the inversion effect context and at cortical level, and supports general reliability of the obtained results.

Some insightful considerations can be drawn from the results of the relevance analysis.

First, higher relevance in the right vs. left ROIs confirmed the right lateralization of holistic processing for faces^71^ and also indicated right hemisphere predominance in body processing^12^.

Second, the partial overlap between relevant areas for face and body decoders shows that the processing of these stimuli shares some important holistic mechanisms, but the overlap is not complete (see Ref.^13^ for further information). The right lateral occipital cortex was crucial for both faces and bodies, while the fusiform gyrus for faces only. This confirms the relevance of the OFA and FFA for faces^16^ and EBA for bodies encoding^24^. FBA was not emphasized by our analysis, but this could be related to the fact that this area encodes for body features such as familiarity^27^, which were not processed in our experimental task. High relevance in right precuneus and lingual gyrus also confirms the previous literature as well. The precuneus was already shown to be implicated in face processing^19,72^, and the lingual gyrus was identified in some studies, too^73,74^.

Third, the relevance of right superior and inferior parietal cortices for both face and body inversion decoding represents a very intriguing result. A possible explanation for the relevance of these parietal areas could be the recruitment of additional neural mechanisms for processing inverted face and body stimuli, which may become closer to objects in terms of mechanisms engaged for perception^75^. In particular, the involvement of these ROIs could represent the use of space-oriented attention^76^. Indeed, we could speculate that mental rotation processes are required to process inverted faces and bodies, and these processes typically involve the right parietal lobe^77^, implicated in visuospatial transformation and in processing spatial features of objects. Interestingly, for control stimuli (houses), the right inferior parietal cortex appeared as the only relevant area for orientation discrimination (see Fig. 5c), although in a different time window (around 300 ms) compared to face and body. It might be possible that right parietal cortex involvement reflects a more general mechanism for processing rotated orientation, which however takes place in different time windows for social stimuli (as early as 150–200 ms) than for non-social stimuli (300 ms). Other different interpretations of the results concerning parietal cortex relevance are possible too. Indeed, a study by Hodzic et al.^27^ found the existence of a third region (beside the previously reported EBA and FBA) in the inferior parietal lobe that might be sensitive to the perception of human bodies. This region seems to be involved in the identification of both body parts and whole bodies^27^ and could play a role also in face perception, according to our results. In fact, we previously found that parietal EEG activity (in theta-band oscillations) mediates the face-inversion effect^35^, which was explained as feature-based attention towards inverted faces. Moreover, we should also consider that the inferior parietal lobe is part of the mirror network^78^. As such, the correct (holistic) processing of upright faces and bodies could activate the mirror neurons of human participants, as opposed to when this processing is disrupted by inversion. Finally, it is worth mentioning that inferior parietal cortex has been found to have a role in temporal predictability, especially in case of fixed temporal expectations^79^. Trials in our experimental paradigm started with an interval with fixed duration (1 s), during which subjects gazed to a fixation cross, making the stimulus onset highly predictable. As this interval was used as baseline correction, it is possible that expectation effect in the baseline interval has been transferred to the subsequent post-stimulus interval, altering the activation level of expectation-sensitive brain regions, such as the inferior parietal cortex^79^. However, we deem improbable that our results about right inferior parietal involvement may reflect this expectation effect since our analysis evidences differential changes between experimental conditions (all affected by the same expectation effect) and since mainly the left rather than the right parietal cortex was observed to be implicated in fixed temporal prediction^79^. Of course, all these interpretations remain speculative at this stage, and the findings about possible involvement of parietal cortices in social stimuli processing need to be considered as preliminary results that warrant further investigation via ad-hoc fine-tuned experiments aimed to clarify the role of these regions in visuospatial attention and mental rotation in social and non-social stimuli processing.

Lastly, overall the relevance analysis provided lower values in case of body stimuli compared to face stimuli, indicating that neural activity had slightly less power in discriminating body orientation (emerging also from the slightly but not significantly lower performance for the body vs face decoders). This might suggest that faces rely on holistic processing more than bodies, which is disrupted by inverted orientation, and therefore neural activity reflects this alteration to a larger extent in case of faces than bodies.

The activity of the brain areas most relevant for decoding was modulated by stimuli orientation both in time domain (more pronounced and later N170 for inverted stimuli) and time–frequency domain (increased ERS in theta-band for inverted stimuli). Theta ERS is likely related to both evoked and induced activity, especially in case of faces. In line with previous observations^34^, differences in neural activity between inverted vs. upright stimuli resulted larger in case of faces than bodies, further suggesting that, although body and face perception share similar mechanisms, quantitative differences exist between them.

The types of modulations observed here were already observed at the scalp level for both face and body processing^35^, thus confirming, at the cortex level, the influence of low-frequency oscillations which are known to be coupled with the (most renowned) gamma-band oscillations in social stimuli processing^80^. It is worth noticing that our results did not highlight significant differences in gamma-band oscillations. This could be related to the high inter-subject variability in gamma-band latency and power in face processing^81^. Indeed, in the proposed framework, the neural decoders were trained and evaluated by adopting a leave-one-subject-out strategy, to highlight robust and shared cross-subject EEG features related to inversion. Therefore, our framework could have missed features that show a high inter-subject variability, focusing only on features globally shared across subjects. To better describe neural features that present a high inter-subject variability, subject-specific (and not cross-subject as in this study) decoders need to be trained. However, subject-specific training requires datasets having more recorded trials per participant (e.g., approximately > 200 total trials per participant^45^) than the one used in the present study (about 30 trials per participant).

Of course, the study has some limitations, and directions for future investigations can be outlined. One limitation concerns the use of a template head model, rather than individual head models, to estimate cortical source activities from EEG signals (a limitation common to a large body of EEG literature, especially in case of healthy participants). However, here we did not focus on activity of single voxel or of very small areas, rather we considered overall regions of interest and their representative activity. Therefore, we expect that some spatial inaccuracies in source reconstruction might have been mitigated by the adopted approach; reassurances come from the obtained results that identified relevant face and body ROIs in agreement with existing literature.

Another possible limitation could be related to the adopted CNN architecture. This was inspired by an architecture widely used for EEG decoding at scalp level (EEGNet^52^), with some modifications introduced to increase accuracy performance compared to the original design, by performing empirical evaluations during preliminary analyses (not shown here). A more complete approach could use automatic techniques to optimize the network architecture specifically for cortex-level decoding, such as Bayesian optimization.

As already highlighted previously, here we used a cross-subject strategy to train the decoders, due to the reduced number of trials per participant. While this choice provides more robust results, it hinders the possibility to investigate neural features at a single-subject level, discovering individual differences, other than shared features, and to possibly evidence clusters of participants characterized by different neural strategies. This kind of analysis would be of great relevance to couple subject-specific differences in EEG discriminant activity with individual differences in behavioral face/body processing. Indeed, as suggested by Yovel et al.^82^ individual differences in face (and body) processing can provide valuable information to extend group-level analysis. This interesting issue can be addressed in future studies, acquiring a larger number of trials per participant, and possibly even enlarging the number of participants. In particular, this may also be of relevance to verify our provisional result showing involvement of parietal cortices.

Despite these desired improvements, the proposed approach based on explainable artificial intelligence, appears promising as a data-driven analysis tool to investigate, at a cortex level, neural features underlying perceptual task. Subsequent studies may use this same approach to explore other perceptual phenomena related to body and face perception, such as perception of whole bodies vs. single parts, perception of static vs. moving bodies, recognition of facial emotions, or other perceptual phenomena in different contexts (e.g., multisensory vs. unisensory perception) or even movement-related phenomena. Furthermore, while in the present study, deep learning decoders and explanation techniques were applied to ROI cortical activities, in future works also cortical connectivity among ROIs, e.g., estimated via Granger Causality, could be analyzed with these techniques.

## Conclusions

In conclusion, our study shows that an artificial intelligence approach, using CNN-based decoders paired with an explanation technique, can be usefully adopted to investigate face and body inversion effects at the cortical level. The CNN decoders, fed with cortical signals reconstructed from EEG at all ROIs (covering the entire cortex) and at all time samples (entire epoch), were able to discriminate the orientation of body and face stimuli. The explanation technique explained network decision in terms of a reduced collection of input features (signals at a few cortical ROIs and a specific time interval) identified as the most relevant in driving the classification output. This suggests a differential engagement of the so identified ROIs in holistic vs. non-holistic processing of face and body stimuli when presented upright vs. inverted, early after stimulus presentation (150–200 ms). The cortical activity of these ROIs exhibited typical larger and later N170 peak, and increased theta activity in case of inverted vs. upright stimuli. The identified ROIs, mainly in the right hemisphere, were only partially overlapped between face and body, and some of them were already known to be specific for body perception and face perception, with right lateral occipital cortex resulting common to face and body, and fusiform gyrus, lingual gyrus and precuneus being distinctive for face. Moreover, as a novel result, the approach pointed to two right parietal areas (superior parietal and inferior parietal cortex) as possibly involved both in face and body inversion effect. It is possible that the adopted method, maximally exploiting class-specific discriminant features contained in the data, was capable to uncover the role of ROIs previously unexplored or not identified as relevant. The involvement of these parietal ROIs could reflect additional non-holistic mechanisms (less body- and face-specific and more object-general, e.g., visuospatial attention and mental rotation mechanisms) engaged to processing inverted stimuli when the holistic processing is broken down or may represent other mechanisms involved in holistic processing and disrupted by inversion. These preliminary findings about parietal cortices may inspire future experiments specifically aimed to clarify the role of these regions in holistic vs. more general visuospatial mechanisms.

Overall, the proposed approach looks promising for investigation of cortical features, reconstructed from EEG, underlying perceptual (or even motor) phenomena, and this study may represent a stimulating starting point for this line of research.

## Supplementary Information

Supplementary Information.

## Data Availability

The datasets used in this study are available on request submitted to the authors Francesco Bossi (francesco.bossi@imtlucca.it) and Davide Rivolta (davide.rivolta@uniba.it).
