# Supplementary material for: Deep learning applied to EEG source-data reveals both ventral and dorsal visual stream involvement in holistic processing of social stimuli
Source: Sci Rep. 2023 May 5;13:7365. doi: 10.1038/s41598-023-34487-z (PMC10162973; doi:10.1038/s41598-023-34487-z)
Supplement: Supplementary file 1 — Supplementary Information. [file 41598_2023_34487_MOESM1_ESM.pdf]

SUPPLEMENTARY INFORMATION

Deep learning applied to EEG source-data reveals both ventral and dorsal visual stream involvement in holistic processing of social stimuli

Davide Borra[1§] (0000−0003−3791−8555)
Francesco Bossi[2§] (0000-0003-3359-8187)
Davide Rivolta[3†] (0000-0002-9969-9135)
Elisa Magosso[1,4†*] (0000−0002−4673−2974)

[1]Department of Electrical, Electronic and Information Engineering "Guglielmo Marconi" (DEI), University of Bologna, Cesena Campus, Cesena, Italy
[2]MoMiLab Research Unit, IMT School for Advanced Studies Lucca, Lucca, Italy
[3]Department of Education, Psychology, and Communication, University of Bari Aldo Moro, Bari, Italy
[4]Alma Mater Research Institute for Human-Centered Artificial Intelligence, University of Bologna, Bologna, Italy

§Authors with shared first authorship
[†]Authors with shared senior authorship

*** Corresponding Author**
Elisa Magosso
e-mail: elisa.magosso@unibo.it

Section S1. Convolutional neural network

In Table S.1, details about the parameters defining the network (i.e., hyper-parameters), together with the number of parameters to fit (i.e., "trainable" parameters) introduced by each layer and the shape of layer outputs, are reported.

Table S1 – Details of the convolutional neural network. Each layer is provided with its name, main hyper-parameters, number of trainable parameters and output shape. Where not specified, stride (S) and padding (P) were set to $(1,1)$ and $(0,0)$, respectively. The total number of trainable parameters was 1502.

Layer name	Hyper-parameters	No. of tr. parameters	Output shape
Input		0	(1, 68, 100)
Conv2D	$K_0 = 4, F_0 = (1,49), P_0 = (0,24)$	196	(4, 68, 100)
BatchNorm2D		8	(4, 68, 100)
Depthwise-Conv2D	$D_1 = 2, K_1 = K_0 \cdot D_1 = 8, F_1 = (68,1)$	544	(8, 1, 100)
BatchNorm2D		16	(8, 1, 100)
ReLU		0	(8, 1, 100)
AvgPool2D	$F_p = (1,10), S_p = (1,2)$	0	(8, 1, 46)
Dropout	$p = 0.25$	0	(8, 1, 46)
Flatten		0	(368)
Fully-Connected	$N_c = 2$	738	(2)
Softmax		0	(2)
		1502	

The input layer simply replicates the input neural activity in a single feature map; thus, the output shape of this layer is (1,68,100). Then, the first convolutional layer performs 2-D convolution in the temporal domain using $K_0 = 4$ temporal kernels with size $F_0 = (1,49)$ (thus, capturing frequency information at 4 Hz and above [1]), unitary stride and zero-padding such that the local output shape matches the input shape, i.e., $P_0 = (1,24)$. Neuron activations were then normalized via batch normalization [2]. The second convolutional layer performs 2-D depthwise convolution [3] in the spatial domain, learning a set of $D_1 = 2$ spatial kernels for each filtered version of the input (8 in total, as $K_0 = 4$), with size $F_1 = (R, 1) = (68,1)$ (that is, learning the optimal combination across all ROIs), unitary stride and no padding. Neuron activations were then normalized via batch normalization, passed through a ReLU non-linearity, and downsampled in time using an average pooling layer with pooling size $F_p = (1,10)$ and pooling stride $S_p = (1,2)$, which is equivalent to applying a moving average in the time-axis within windows of 5 ms with a step of 1 ms. Then, neuron activations were dropped out during training using a dropout probability of $p = 0.25$. The output neuron activations were then flattened into a 1-D array and provided as input to the fully-connected layer with $N_c = 2$ neurons, providing the class scores $o_k, k = 0,1$ as output. Finally, class scores were converted into the conditional probabilities by using the softmax activation function.

By keeping limited the number layers and of learned features (e.g., only 4 temporal kernels and 16 spatial kernels, overall) and by including depthwise convolutions, which are convolutions specifically aimed to reduce the number of trainable parameters, the adopted

CNN introduced only 1502 parameters (contained in θ). To optimize the trainable parameters in θ, the cross-entropy between the empirical probability distribution (defined by training labels) and the model probability distribution (defined by CNN outputs) was used as loss function and it was minimized using the Adaptive moment estimation (Adam) algorithm [4] with a mini-batch size of 64, learning rate of 10^{-4} and other parameters set as in its default implementation [5]. CNNs were trained for 500 epochs and the training was stopped when the validation loss did not decrease for 100 consecutive epochs (this parameter was based on the convergence speed of the algorithm via empirical evaluations), as also performed in [6,7].

The main hyper-parameters (e.g., the number of temporal kernels, temporal kernel size, the number of spatial kernels, and the pooling size and stride) were selected via empirical evaluations during preliminary analyses.

CNNs were developed in Python (version 3.8.10) and trained with PyTorch (version 1.9.0) [5] and network decisions were explained with Captum (0.5.0) [8], using a workstation equipped with an AMD Threadripper 1900X, NVIDIA TITAN V and 48 GB of RAM.

Section S2. Layer-wise relevance propagation

Layer-wise relevance propagation [9] propagates the prediction of the network, represented by the class score o_k (e.g., the predicted score associated by the network to the inverted orientation, o_1, see Eq. 2), backward in the network. To do so, propagation rules must be defined for each layer of the network. Let m and n be the indices of two neurons of two consecutive layers ($l-1$ and l) of the network and let $R_n^{(l)}$ be the relevance for the neuron n of the layer l in predicting o_k. The backward propagation of the relevance at a given layer back to a preceding layer of the network is achieved by applying the rule:

$$R_m^{(l-1)} = \sum_n \frac{z_{mn}}{\sum_m z_{mn}} R_n^{(l)}, \tag{B.1}$$

where z_{mn} weights how much the neuron m contributed to make the neuron n relevant, and the denominator $\sum_m z_{mn}$ forces the conservation of the relevance during the propagation. Indeed, the conservation is ensured locally by $\sum_m R_m^{(l-1)} = \sum_n R_n^{(l)}$, and thus, globally throughout the network, as $\sum_i R_i^{(0)} = \cdots = \sum_n R_n^{(l)} = \cdots = o_k$.

The propagation rule applied in this study is the LRP-ε rule [10]:

$$R_m^{(l-1)} = \sum_n \frac{a_m w_{mn}}{\varepsilon + \sum_m a_m w_{mn}} R_n^{(l)}, \tag{B.2}$$

where the term a_m denotes the activation of the neuron m, w_{mn} denotes the weight of the connection from unit m to unit n, and ε is a small positive term that ensures that $R_m^{(l-1)}$ is bounded for small or null values of neuron activations in the denominator $\sum_m a_m w_{mn}$.

References

1. Lawhern, V. J. *et al.* EEGNet: a compact convolutional neural network for EEG-based brain–computer interfaces. *Journal of Neural Engineering* **15**, 056013 (2018).
2. Ioffe, S. & Szegedy, C. Batch Normalization: Accelerating Deep Network Training by Reducing Internal Covariate Shift. in *Proceedings of the 32nd International Conference on Machine Learning* (eds. Bach, F. & Blei, D.) vol. 37 448–456 (PMLR, 2015).
3. Chollet, F. Xception: Deep Learning with Depthwise Separable Convolutions. *2017 IEEE Conference on Computer Vision and Pattern Recognition (CVPR)* 1800–1807 (2016).
4. Kingma, D. P. & Ba, J. Adam: A Method for Stochastic Optimization. *arXiv:1412.6980 [cs]* (2017).
5. Paszke, A. *et al.* Automatic differentiation in PyTorch. in *NIPS-W* (2017).
6. Borra, D. & Magosso, E. Deep learning-based EEG analysis: investigating P3 ERP components. *Journal of Integrative Neuroscience* **20**, 791–811 (2021).
7. Borra, D., Fantozzi, S. & Magosso, E. A Lightweight Multi-Scale Convolutional Neural Network for P300 Decoding: Analysis of Training Strategies and Uncovering of Network Decision. *Frontiers in Human Neuroscience* **15**, 655840 (2021).
8. Kokhlikyan, N. *et al.* Captum: A unified and generic model interpretability library for PyTorch. Preprint at http://arxiv.org/abs/2009.07896 (2020).
9. Bach, S. *et al.* On Pixel-Wise Explanations for Non-Linear Classifier Decisions by Layer-Wise Relevance Propagation. *PLoS ONE* **10**, e0130140 (2015).
10. Montavon, G., Binder, A., Lapuschkin, S., Samek, W. & Müller, K.-R. Layer-Wise Relevance Propagation: An Overview. in *Explainable AI: Interpreting, Explaining and Visualizing Deep Learning* (eds. Samek, W., Montavon, G., Vedaldi, A., Hansen, L. K. & Müller, K.-R.) vol. 11700 193–209 (Springer International Publishing, 2019).
